# Hydroclimate, Thallus Dynamics and Multi-Seasonal Reproductive Phenology in *Mannia triandra*

**DOI:** 10.3390/plants15182808

**Published:** 2026-09-13

**Authors:** Michaela Kropik, Anna-Lena Hauser, Harald G. Zechmeister

**Affiliations:** Biodiversity Dynamics and Conservation Group, Department of Botany and Biodiversity Research, University of Vienna, Rennweg 14, 1030 Vienna, Austria; lena@d-hauser.com (A.-L.H.); harald.zechmeister@univie.ac.at (H.G.Z.)

**Keywords:** *Mannia triandra*, bryophyte phenology, reproductive phenology, thallus dynamics, microclimate, poikilohydry, vapour pressure deficit, hydroclimate

## Abstract

Poikilohydric plants are closely coupled to atmospheric moisture, but their responses to hydroclimate may differ among life cycle phases. We investigated vegetative and reproductive dynamics of the thallose liverwort *Mannia triandra* in Gesäuse National Park, Austria, using surveys at approximately 14-day intervals over one annual cycle in two quantitatively monitored populations, combined with continuous near-surface temperature and relative humidity measurements. We quantified relative thallus area change, frost exposure, carpocephalum emergence, and post-emergence development. At the site × survey interval scale, median relative thallus area change declined with increasing 14-day mean vapour pressure deficit (standardised β = −0.58, 95% CI [−1.00, −0.16]), whereas temperature had no independent association. Cold-season dynamics did not support a simple frost damage response: longer frost duration was not associated with greater thallus loss, and frost exposure did not increase the probability of negative area change. Reproductive development was multi-seasonal. Carpocephala occurred on thalli persisting from the previous growing season, newly recorded carpocephala were concentrated in early spring, and subsequent development progressed seasonally towards maturation and dehiscence. These findings suggest a potential carry over across life cycle phases: short-term atmospheric drying was reflected in vegetative thallus dynamics, while subsequent reproductive development occurred on gametophytic tissue that persisted across seasons.

## 1. Introduction

Phenological shifts are among the most visible biological responses to contemporary climate change, yet most evidence comes from vascular plants [1]. Bryophytes remain comparatively underrepresented in phenological climate-change research, despite their substantial contributions to ecosystem functioning, including water retention, nutrient cycling and carbon dynamics [2,3]. Their water relations differ fundamentally from those of vascular plants: as poikilohydric organisms, their tissue hydration and physiological activity remain closely coupled to ambient moisture conditions and atmospheric moisture demand [4,5]. Bryophyte responses to changing hydroclimate may thus depend on how sensitivity is expressed across the life cycle. Changes in temperature, precipitation and vapour pressure deficit (VPD) may therefore affect both vegetative dynamics and the timing and progression of reproductive development. Hydroclimatic sensitivity may consequently be expressed across multiple components of the bryophyte life cycle.

The close coupling to external moisture is especially important for sexual reproduction. Fertilisation requires free water, and the developing sporophyte remains physiologically dependent on the maternal gametophyte [6,7,8]. Experimental evidence also supports generation-specific sensitivity. In desert mosses, reproductive output can be strongly constrained by patch hydration, with hydroperiods often short and seasonally restricted [9]. Experimental evidence further indicates generation-specific stress sensitivity: sporophytes may be more vulnerable than maternal gametophytes to rapid desiccation [10] and thermal stress [11].

Climate effects on reproduction may therefore arise not only during visible sporophyte maturation but also through earlier phases of gametophyte persistence, gametangial development and fertilisation. Consistent with this, bryophyte reproductive phenophases, including sex expression, gametangial maturation and sporophyte development, have been linked to seasonal transitions between wet and dry periods in tropical and temperate systems [12,13,14]. Understanding bryophyte responses to changing hydroclimates therefore requires a life cycle perspective that links visible reproductive outcomes to earlier developmental and vegetative phases.

This life cycle perspective is needed because bryophyte phenology is inherently structured by distinct phenophases, spanning vegetative development, sex expression, fertilisation, sporophyte development and spore release. Some stages may be completed within days, whereas gametangial initiation, arrested sporophyte development and capsule maturation can extend over several months or across seasons [15,16]. Early microenvironmental phenology studies further showed that vegetative dynamics, gametangial formation, fertilisation and sporophyte development may be associated with different combinations of moisture conditions, vapour pressure deficit, temperature, light and day length [17,18,19].

Hydroclimatic sensitivity may therefore differ among phenophases rather than being expressed uniformly across the life cycle. Long-term field studies support this view: bryophyte sporophyte production, abortion and spore release can reflect moisture conditions acting during earlier phases of gametangial development, fertilisation and sporophyte maturation, rather than only conditions at the time of dispersal [9,14,20,21,22]. Recent long-term airborne-eDNA evidence further suggests that bryophyte spore phenology is shifting towards earlier and longer dispersal seasons under contemporary climate change [23]. From a functional-trait perspective, however, life cycle stage, reproductive traits, water relations and measurement scale remain poorly integrated in bryophyte ecology [24]. Field studies resolving multiple bryophyte phenophases against continuous in situ microclimate are therefore still rare.

A further challenge is scale. For bryophytes, the relevant climatic environment is often the near-surface microclimate experienced by the thallus, rather than the macroclimate recorded by distant weather stations. Boundary-layer conditions, shading, water films, snow cover and evaporative demand can vary over very small spatial scales, shaping hydration, desiccation risk, frost exposure and reproductive development [5,25,26]. Macroclimatic datasets may therefore obscure the environmental variation most directly relevant to bryophyte performance. Building on earlier microenvironmental phenology work [17,18,19], field studies that combine repeated observations across multiple bryophyte life cycle phases with continuous in situ microclimate are needed to identify when hydroclimatic sensitivity is expressed under natural field conditions.

To address this gap, we studied the thallose liverwort *Mannia triandra* (Scop.) Grolle, a species of Community interest listed in Annex II of the EU Habitats Directive, in Gesäuse National Park, Austria. We combined repeated plot-scale surveys across one annual cycle with continuous in situ microclimate records to examine how hydroclimatic coupling is expressed in short-term vegetative thallus area dynamics, cold-season thallus persistence and reproductive development.

First, we tested whether net vegetative thallus area dynamics were associated with short-term hydroclimate. We predicted that relative thallus area change would be lower after two-week periods of higher vapour pressure deficit (VPD), reflecting greater atmospheric moisture demand. For cold-season intervals, we tested whether area loss increased with frost exposure, as expected if frost acted as a direct damage driver; freeze–thaw frequency was considered as an exploratory indicator of frost-regime instability.

Second, we assessed whether spring reproductive expression was associated with previous-season thallus state. We predicted that subplots with greater previous-season thallus expansion would be more likely to produce newly recorded carpocephala, while treating subplot-level thallus area as a coarse proxy for the condition of individual reproductive thalli.

Third, we examined the seasonal timing of reproductive emergence and post-emergence carpocephalum development across the annual monitoring period. We expected carpocephala to appear within a restricted seasonal window, followed by a seasonally structured progression of carpocephalum elongation and maturation.

## 2. Results

### 2.1. Vegetative Thallus Area Change Declined After Intervals with Higher VPD

**Figure 1:**
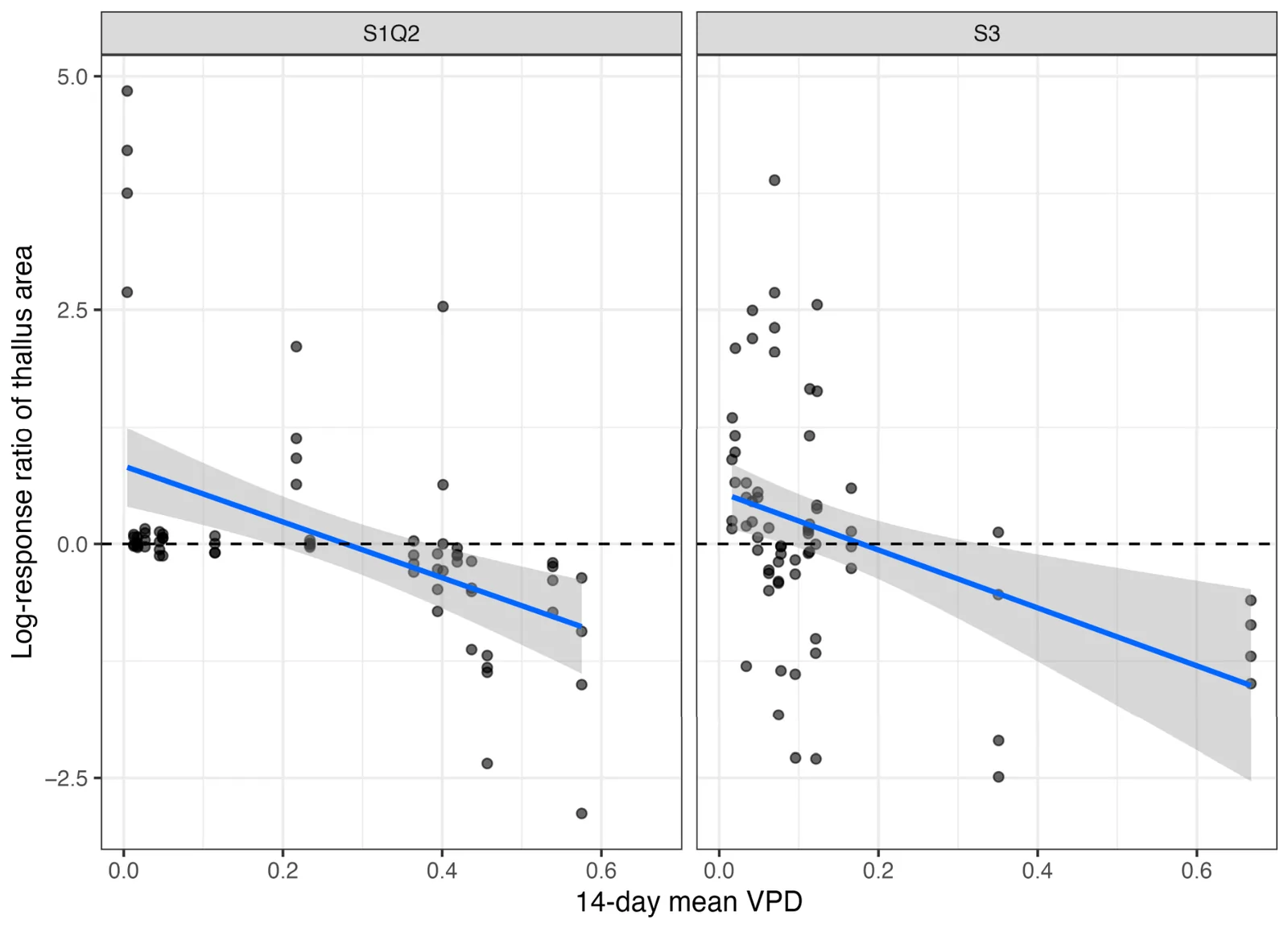
Relationship between relative thallus area change, expressed as log-response ratio, and 14-day mean vapour pressure deficit at the two quantitatively monitored sites. Lines show site-specific linear fits for visualisation only. Grey shaded bands indicate 95% confidence intervals. Both visual fits were negative, suggesting that the overall VPD association was not attributable to one monitored site alone.

Relative thallus area change, aggregated as the median log-response ratio at the site × survey interval scale, decreased with increasing vapour pressure deficit (VPD; standardised predictor; β = −0.58, 95% CI [−1.00, −0.16], *p* = 0.008; Table 1, Figure 1). Temperature showed no independent association once VPD was included (β = 0.09, 95% CI [−0.33, 0.51], *p* = 0.680). The analysis included 34 site × survey interval observations.

The negative VPD association was also retained in the subplot-level model (β = −0.61, 95% CI [−0.85, −0.37], *p* < 0.001; *n* = 136 subplot-interval observations; Table S2). The same qualitative pattern was obtained when mean rather than median relative area change was used, after excluding observations with very small previous thallus areas, in an alternative area-state model, and when site identity was included as an additional fixed effect (Tables S2 and S3). Model diagnostics indicated moderate heteroscedasticity at higher fitted values and minor tail deviations in Q–Q plots (Figure S1), but the main VPD association was consistent across sensitivity analyses.

### 2.2. Cold-Season Thallus Area Dynamics Do Not Support a Simple Frost Damage Response

Cold-season thallus dynamics did not show the pattern expected under a simple frost damage interpretation. In an exploratory subplot-level analysis of frost-exposed cold-season intervals, relative thallus area change did not become more negative with increasing cumulative frost exposure. Instead, the estimated association was positive (β = 0.29, 95% CI [0.01, 0.58]; *n* = 24 subplot-interval observations; Table 2). However, influence analyses indicated that the estimate was sensitive to influential observations (Table S4). We therefore do not interpret the positive coefficient as evidence for a beneficial effect of frost. Rather, the analysis did not support the predicted increase in thallus loss with longer frost exposure.

The same qualitative pattern was obtained in exploratory sensitivity analyses using frost days and minimum temperature as alternative cold-exposure metrics (Table S5). In a complementary subplot-level binomial model including intervals with and without frost, cumulative frost exposure did not show an increased probability of negative relative thallus area change (β = −0.12, 95% CI [−0.55, 0.25]; *n* = 112 subplot-interval observations; Table S6). Freeze–thaw frequency was limited and unevenly distributed across frost-exposed intervals and showed no clearly interpretable relationship with relative thallus area change.

Field observations indicated snow cover at the monitored plots during two cold-season periods, from 20 November to 6 December 2023 and from 20 January to 1 February 2024. Frost exposure inferred from air-temperature records therefore describes near-surface thermal conditions and may not fully represent the direct freezing stress experienced by thallus tissue during periods of snow cover.

### 2.3. Previous-Season Thallus State Did Not Clearly Predict Spring Reproductive Expression

Across the eight monitored subplots, spring reproductive expression showed no clear positive relationship with previous-season thallus state. Newly recorded carpocephala occurred across a broad range of previous-season thallus state values and were absent from some subplots with intermediate values.

**Figure 2:**
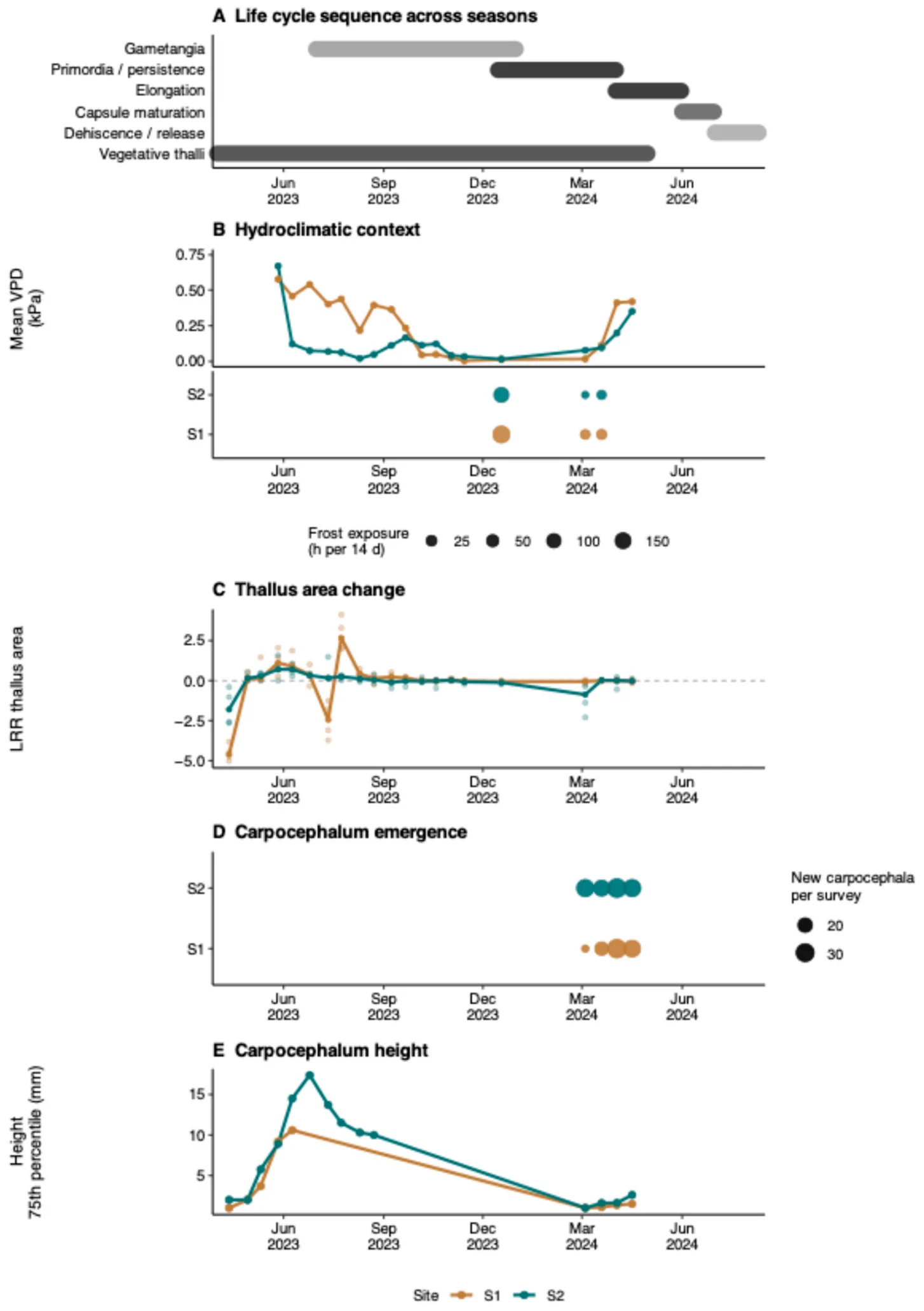
Annual life cycle dynamics and hydroclimatic context of *Mannia triandra* in two quantitatively monitored populations in Gesäuse National Park, Austria. In panels B–E, colours distinguish the two monitored sites (S1 and S2). (**A**) Schematic summary of the observed multi-season reproductive sequence. Bars indicate the approximate timing of vegetative thalli, gametangial development, carpocephalum primordia and cold-season persistence, carpocephalum elongation, capsule maturation and dehiscence/spore release. (**B**) Local hydroclimatic context during the monitoring period, showing 14-day mean vapour pressure deficit (VPD) and cumulative frost exposure during the 14-day windows preceding surveys. Frost points are shown only when frost occurred during the preceding 14-day interval; point size indicates cumulative frost hours. (**C**) Relative vegetative thallus area change between consecutive surveys, expressed as log-response ratios. Points show subplot-level observations and lines show site-level medians. (**D**) Newly recorded carpocephala from the 2024 cohort, summarised at the site × survey-date level. Point size indicates the number of newly recorded carpocephala. (**E**) Post-emergence carpocephalum development across the monitoring period, shown as the site-level 75th percentile of measured carpocephalum height (mm) where carpocephala were present. Height measurements include late development of the 2023 cohort already present at the start of monitoring and the subsequent 2024 cohort. The figure summarises descriptive repeated-measures dynamics within the two monitored populations.

Newly recorded carpocephala were temporally concentrated between early March and early April at both monitored sites (Figure 2D). Survey intervals with and without newly recorded carpocephala spanned a similar range of preceding VPD conditions, indicating no clear short-term VPD signal within the observed spring window. Spring reproductive expression was therefore temporally clustered in early spring rather than distributed throughout the full observation window or tightly aligned with short-term variation in atmospheric moisture demand.

### 2.4. Reproduction Followed a Multi-Seasonal Sequence

Field observations revealed a structured reproductive sequence spanning multiple seasons. Gametangia developed on green thalli from mid-summer onwards. Archegonia were first observed in July and August, whereas antheridia appeared later, from late August onwards. The main period of gametangial maturation occurred in late autumn, with both male and female gametangia commonly mature in November and December.

Early carpocephalum primordia became visible around mid-December, after the late-autumn period of gametangial maturation. Developing carpocephala remained sessile throughout winter and early spring and began to elongate in early April. Fully expanded carpocephala were typically observed in May, and sporophyte maturation occurred from early to late June, with timing differing between sites. Mature capsules began to dehisce from early July onwards, when carpocephala and supporting thalli were already visibly senescent. Apparent spore release was observed from early July onwards. By early August, only remnants of brown carpocephala remained.

Across all observations, carpocephala were associated with thalli originating from the previous growing season. These observations indicate that reproductive development was carried by gametophytic tissue that had persisted across seasons, rather than by thalli newly produced during the same spring.

### 2.5. Post-Emergence Carpocephalum Development Followed Seasonally Structured Trajectories

Carpocephalum height showed pronounced seasonal trajectories at both monitored sites, with low values during winter and early spring, rapid elongation during spring, and maximum values in early summer (Figure 2E). Because elongation occurred during the warmer and drier part of the season, periods of elevated carpocephalum height overlapped with seasonally higher VPD. This temporal overlap was not interpreted as a direct VPD effect.

Developmental trajectories differed between sites. At S1, carpocephalum development was concentrated within a narrower seasonal window and reached a pronounced early-summer peak. At S2, elevated carpocephalum heights persisted across a broader range of survey dates and VPD conditions. Because seasonal progression, atmospheric drying and site identity were closely aligned in the field data, these site-specific patterns are interpreted descriptively rather than as evidence for a single causal driver.

Field observations provided little evidence for widespread sporophyte abortion before maturity. Most observed carpocephala appeared to progress to maturity and subsequently senesced or disappeared after capsule dehiscence. This pattern is interpreted descriptively as a cohort-level observation rather than as an estimate of individual sporophyte survival.

## 3. Discussion

High-resolution, microclimatically informed monitoring of *M. triandra* showed that hydroclimatic associations differed among life cycle phases. Vegetative thallus dynamics were most clearly associated with atmospheric drying, with relative thallus area change declining after two-week periods of higher vapour pressure deficit (VPD). In contrast, exploratory cold-season analyses did not show the increase in thallus loss expected under a simple frost damage hypothesis. Reproductive expression and sporophyte development followed a multi-seasonal developmental sequence, with no clear short-term VPD signal apparent within the observed reproductive window. Together, these findings support a phase-specific view of hydroclimatic sensitivity in *M. triandra*.

Viewed from a bryophyte life-history perspective, this phase-specific pattern is broadly consistent with aspects of a short-lived shuttle strategy sensu During [27], combining conspicuous sporophyte production with gametophyte persistence through at least one unfavourable period. In *M. triandra*, sporophytes developed only on thalli that had persisted from the previous growing season, suggesting that reproduction was embedded in a multi-seasonal sequence rather than a single short favourable window.

### 3.1. Net Thallus Area Change Was Lowest After Higher-VPD Intervals

The clearest hydroclimatic signal was the negative association between VPD and relative thallus area change. This is consistent with the poikilohydric biology of bryophytes, in which tissue water status and activity depend strongly on external moisture conditions [5,28]. Higher VPD increases atmospheric moisture demand and can shorten hydration periods, potentially reducing the time available for carbon gain and visible thallus area expansion. In our models, VPD was more consistently associated with thallus dynamics than air temperature, supporting its interpretation as a functionally relevant predictor of short-term desiccation risk.

Importantly, the negative VPD association was detected in the primary scale-matched analysis, in which relative thallus area change was aggregated to the site × survey interval scale. The same qualitative association was retained in the subplot-level sensitivity analysis, after excluding observations with very small previous thallus areas, in an alternative area-state model, and when site identity was included as a fixed effect. Substantial variation around the relationship is biologically plausible because visible thallus area is a composite field response integrating growth, shrinkage, senescence, hydration state and fine-scale microhabitat heterogeneity. We therefore interpret the VPD association as a shift in net thallus area dynamics during drier periods, not as a direct estimate of growth reduction.

### 3.2. Cold-Season Thallus Dynamics Do Not Indicate Simple Frost Damage

Cold-season thallus dynamics did not show the pattern expected under a simple frost damage hypothesis. If frost exposure were the main driver of cold-season area loss, longer frost duration should have been followed by stronger declines in thallus area. This pattern was not apparent in the exploratory frost analyses. Across intervals with complete frost-exposure summaries, frost exposure did not show an increased probability of negative area change, and alternative frost metrics did not reveal a clearer damage pattern. Within frost-exposed cold-season intervals, the frost-duration estimate was positive but influence-sensitive and was therefore not interpreted as evidence for a beneficial effect of frost. Because frost exposure was shared among subplots within each site × survey interval, we interpret these findings as exploratory evidence from the monitored populations rather than as an independently replicated test of site-level frost effects.

Field observations indicated snow cover from 20 November to 6 December and from 20 January to 1 February. Snow can buffer near-surface conditions against temperature fluctuations, wind, radiation and evaporative demand [29,30], meaning that longer frost duration need not imply greater thallus damage. Such winter buffering may also matter biologically for bryophytes: in *Racomitrium lanuginosum*, snow cover was linked to the maintenance of reproductive phenology, whereas exposed alpine conditions were associated with restricted sporophyte maturation and reduced male gametangial development [31,32]. Because snow cover and cold-season surface conditions were not continuously quantified at the plot scale, this explanation remains tentative. Frost exposure inferred from air temperature alone may therefore have been a poor proxy for damaging winter conditions at the thallus surface in our field setting.

### 3.3. Reproduction Was Multi-Seasonal and Seasonally Structured

Reproductive development differed from the short-term vegetative response to atmospheric drying. Observed carpocephala developed on thalli originating from the previous growing season, indicating that reproduction involved carry over across seasons. This carry over, however, was not expressed as a simple positive relationship between previous-season thallus state and spring reproductive output. Reproduction occurred across a wide range of previous-season thallus state values and was absent from some subplots with intermediate values, suggesting that reproduction in the observed cohort was carried by persistent gametophytic tissue, but that previous-season thallus area was not a sufficient predictor of reproductive expression. This does not exclude resource limitation: bryophyte sporophyte production can carry measurable costs for the gametophyte, and experimental evidence from mosses indicates that successful sporophyte maturation may depend on local gametophytic resources [6,33]. Previous-season thallus area may therefore have been too coarse a proxy for the local gametophytic condition relevant to reproductive expression.

The reproductive sequence was strongly seasonal and multi-stage. Bryophyte phenology studies show that gametangial maturation, fertilisation, sporophyte development and spore dispersal can occupy distinct seasonal windows, with timing differing among species and climates [34,35,36]. In *M. triandra*, gametangial maturation occurred mainly in late autumn, early carpocephalum primordia became visible in winter, carpocephala elongated in spring, and sporophytes matured and dehisced in early summer.

Comparable seasonal structuring of reproduction has been documented in the model liverwort *Marchantia polymorpha* subsp. *ruderalis*. Duckett et al. [37] observed up to three waves of gametangiophore production within a year, with antheridiophores generally preceding archegoniophores by 2–3 months and sporophyte maturation concentrated in summer. This differs from *M. triandra*, where reproductive development extended across successive seasons, from gametangial development in summer and autumn through overwintering of early carpocephalum stages to elongation in spring and sporophyte maturation and dehiscence in early summer. Thus, strongly seasonal reproductive development may take different temporal forms among thalloid liverworts.

The tight seasonal clustering of newly recorded carpocephala is consistent with reproductive expression being seasonally cued. The cues underlying this clustering cannot be identified from our data, but previous studies indicate that bryophyte reproductive timing may respond to photoperiod, rainfall and humidity. Day length can regulate reproductive differentiation in bryophytes [38,39], while liverwort reproductive timing may also be associated with recent rainfall and relative humidity [21]. In *M. triandra*, the persistence of early carpocephalum stages through winter fits a cold-season phenology in which reproductive development may slow before resuming in spring [17,40]. Male expression followed a different seasonal pattern, with antheridia appearing later and overlapping less with the period of strongest summer atmospheric drying. Although its functional significance remains untested here, this pattern is consistent with broader evidence that male gametangial development can be seasonally distinct from female development and sensitive to the length of favourable growth periods or hydroclimatic conditions in bryophytes [14,21,32]. Evidence from water-limited moss systems further suggests that male occurrence and sex expression can decline under more stressful moisture conditions, but whether similar sensitivity occurs in *M. triandra* remains unresolved [36,41].

After emergence, local site context became more apparent. Carpocephalum development was concentrated within a narrower early-summer window at the qualitatively more exposed S1 site, whereas elevated carpocephalum heights persisted longer at the qualitatively shadier S2 site. These differences may reflect local site conditions, including qualitative differences in exposure and shading, but the two-site design does not allow these factors to be separated from site identity. Bryophyte reproduction can be strongly shaped by local microclimatic conditions, even where broader climatic conditions appear unfavourable [42]. In *M. triandra*, field observations provided little evidence for widespread sporophyte abortion before maturity. Most carpocephala appeared to reach maturity before dehiscence, although individual reproductive structures were not permanently marked. This pattern should therefore be interpreted descriptively as a cohort-level field observation rather than as an estimate of sporophyte survival.

### 3.4. A Phenophase-Based View of Bryophyte Hydroclimatic Sensitivity

Our results support considering bryophyte hydroclimatic responses at the level of individual phenophases. In *Mannia triandra*, atmospheric drying was reflected most clearly in short-term vegetative thallus dynamics, whereas exploratory cold-season analyses and reproductive development followed different temporal patterns. This is consistent with bryophyte phenology studies showing that reproductive phases differ in duration and climatic sensitivity and that dispersal may reflect weather conditions acting during earlier stages of gametophyte growth, fertilisation and sporophyte development [21,22].

Such temporal carry over may be relevant under climate change. Long-term airborne-eDNA data indicate earlier and longer bryophyte spore seasons, with preceding-year conditions contributing to spring phenological timing [23]. Although we did not test long-term shifts, the multi-seasonal reproductive sequence observed in *M. triandra* identifies a potential route by which hydroclimatic effects on persistent gametophytic tissue may carry over to subsequent reproduction.

Quantitative monitoring was restricted to two sites and eight subplots, so the observed relationships should not be treated as range-wide estimates. Nevertheless, the study linked continuous near-surface microclimate measurements with an annual sequence of vegetative and reproductive dynamics. Atmospheric drying was associated with lower short-term thallus area change, cold-season dynamics did not support a simple frost damage pattern, and reproduction unfolded across seasons on persistent gametophytic tissue. Together, these findings highlight the value of phenophase-resolved microclimate monitoring for understanding hydroclimatic sensitivity across the bryophyte life cycle.

## 4. Materials and Methods

### 4.1. Study System and Field Design

**Figure 3:**
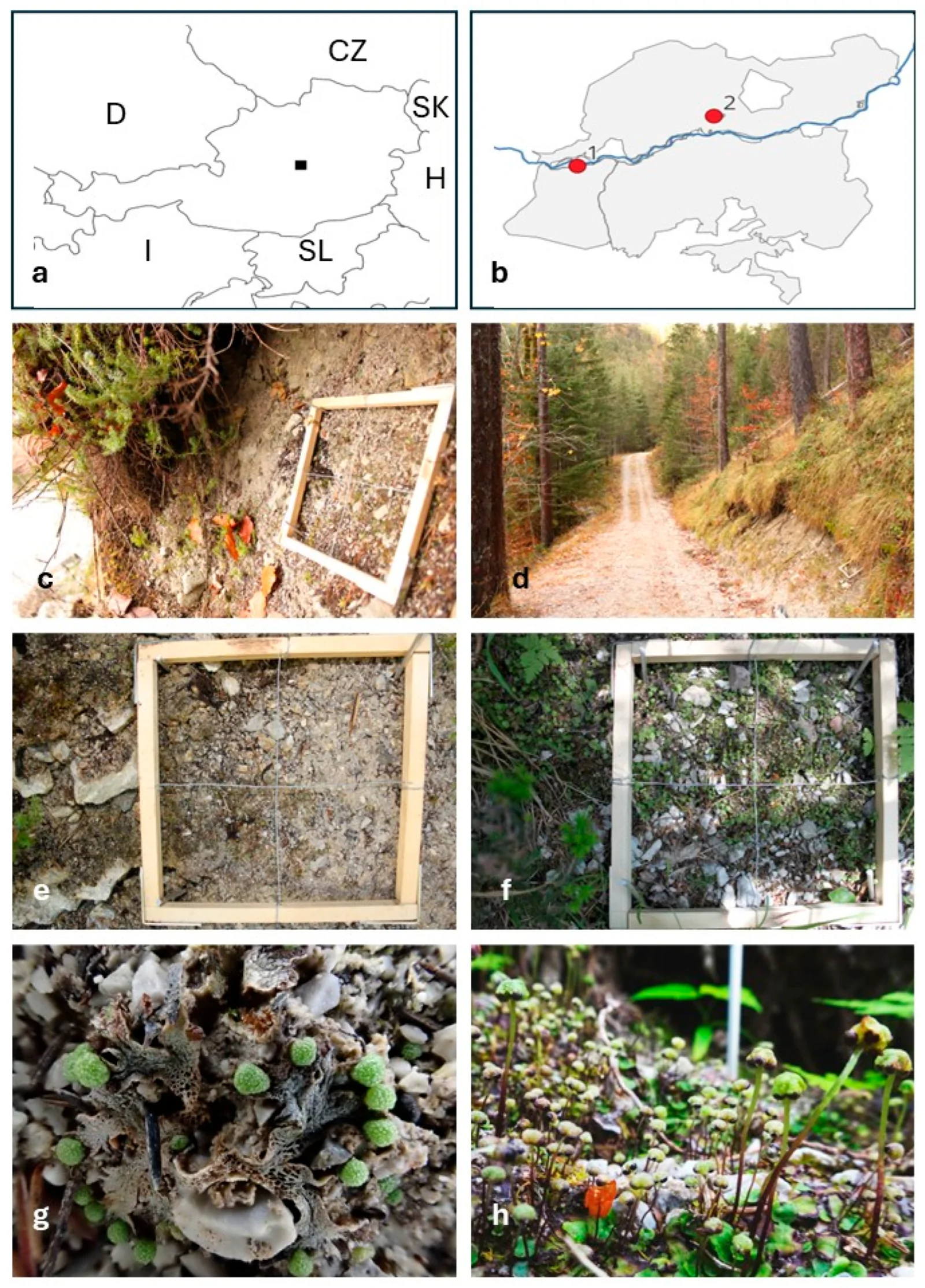
Study area, habitat, field setup, and reproductive structures of *Mannia triandra* in Gesäuse National Park, Austria. (**a**) Location of Gesäuse National Park within Austria, indicated by the black square; (**b**) locations of the two quantitatively monitored populations within Gesäuse National Park, with 1 = S1 and 2 = S2; (**c**,**d**) habitat at S1 and S2, respectively; (**e**,**f**) 20 × 20 cm monitoring frame, divided into four 10 × 10 cm subplots, positioned at S1 and S2, respectively; (**g**) *M. triandra* with elongating carpocephala in April 2024; (**h**) *M. triandra* with carpocephala bearing mature capsules in June 2024.

The study was conducted in Gesäuse National Park, Austria (Figure 3a), an alpine region characterised by steep carbonate massifs and dynamic gravel deposits along river sections with largely natural flow and channel dynamics. The monitored populations of the thallose liverwort *Mannia triandra* (Scop.) Grolle occurred at 600–770 m a.s.l. The broader study region has a mean annual temperature of approximately 8 °C and a mean annual precipitation of approximately 1200 mm.

Four populations of *M. triandra* were monitored. Two populations occurring in semi-caves were excluded from quantitative analyses because limited accessibility prevented standardised repeated surveys. Quantitative analyses were therefore based on the two populations for which standardised spatial monitoring was possible (Figure 3b). These sites were located on calcareous scree slopes along the upper margins of adjacent rock faces. Occupied patches were typically slightly overhung or semi-sheltered (Figure 3c,d). Both sites were sun-exposed but differed in local exposure and shelter, with one site being more open and the other more strongly sheltered. Because this habitat contrast was not replicated, differences between the two sites were interpreted descriptively.

At each site, during the initial survey, a 20 × 20 cm wooden square frame was positioned over the *M. triandra* population to define the area to be monitored. The four corners of the frame were permanently marked with nails, allowing the frame to be repositioned precisely during subsequent surveys (Figure 3e,f). Two wires stretched across the centre of the frame at right angles divided the plot into four 10 × 10 cm subplots, resulting in a total of eight subplots nested within the two sites. The subplots were surveyed in a fixed order: top left, top right, bottom left, and bottom right. This procedure ensured that the same areas of each population were examined consistently throughout the study period. After each survey, the wooden frame was removed and reused at the next site, while the nails remained in place as permanent reference points.

At all four sites, *M. triandra* grew on relatively loose, sandy to gravelly, carbonate-rich mineral substrate rather than organic accumulations, with sparse vegetation cover. No evidence of grazing or other vertebrate-related disturbance was observed at the monitored populations during repeated surveys. At the two quantitatively monitored sites, all accompanying bryophyte and vascular plant species were recorded in each subplot. The following bryophytes were recorded, listed in descending order of frequency: *Marchantia quadrata*, *Encalypta streptocarpa*, *Bryum pallens*, *Fissidens dubius*, *Mesoptychia badensis*, *Tortella tortuosa* s.l., *Weissia* sp., and *Pohlia* sp. Vascular plants were largely absent; *Pinguicula vulgaris* and small individuals of *Asplenium ruta-muraria* occurred in only two subplots. The total cover of all accompanying plant species did not exceed 10% in any subplot, indicating that *M. triandra* occupied predominantly open, sparsely vegetated microsites characterised by largely bare, unconsolidated, carbonate-rich substrate.

Field surveys were conducted at approximately 14-day intervals from April 2023 to April 2024 (25 survey dates), covering one full annual cycle. At each survey, thallus surface area in each subplot was quantified using standardised nadir digital photography (Sony Cyber-shot DSC-WX350, Tokyo, Japan). Images were georeferenced in QGIS (v3.34.1), corrected for geometric distortion, and thallus margins were manually digitised to calculate surface area. Pre-existing, darkened thalli present at the first survey in April 2023 were distinguished from thalli newly established during the 2023 growing season.

Carpocephala were counted at each survey and spatially mapped. To document post-emergence reproductive development, up to ten randomly selected carpocephala per site were measured in situ, where present. Carpocephalum height was measured using a vernier caliper, and developmental stage was classified according to a five-point scale: 0, carpocephala dead or absent; 1, carpocephala sessile; 2, carpocephala extended; 3, capsules green; and 4, capsules open (Figure 3g,h). The presence of gametangia was recorded to document the onset and duration of reproductive development. Their first appearance detectable with a hand lens was recorded, as was their continued presence during subsequent surveys until they were no longer visible.

### 4.2. Microclimatic Measurements, Data Aggregation and Integration

Air temperature (°C) and relative humidity (%) were recorded at each site using DK3xx+ “ruggedPlus” data loggers (Driesen + Kern GmbH, Bad Bramstedt, Germany). Loggers were installed at the substrate surface immediately adjacent to the monitored plots and within the same slightly overhung or semi-sheltered microsites as the thalli. Measurements were recorded at 15 min intervals from May 2023 to April 2024. We therefore treat these records as local near-surface air microclimate, rather than as direct measurements of thallus surface conditions or tissue hydration.

Raw records were screened using conservative plausibility thresholds (temperature < −30 °C or >60 °C, relative humidity outside 0–100%, and VPD > 5 kPa); less than 2% of records were excluded. Vapour pressure deficit (VPD, kPa) was calculated from temperature and relative humidity using the Tetens formulation. Saturation vapour pressure was calculated as es = 0.6108 × exp(17.27 × T/(T + 237.3)), actual vapour pressure as ea = (RH/100) × es, and VPD as es − ea.

To match the approximately 14-day survey interval, microclimatic variables were summarised over the 14 days preceding each survey. Mean VPD over this period was used as the primary measure of atmospheric drying. Analyses based on 14-day microclimatic summaries were restricted to survey dates with complete 14-day records. Climate summaries were matched to biological observations by site and survey date.

### 4.3. Vegetative Thallus Dynamics and Hydroclimate

Quantitative inference was restricted to repeated-measures patterns within the two monitored sites. Microclimatic predictors were measured at the site level and summarised by survey interval, whereas thallus area was recorded for individual subplots. Subplots therefore represent repeated biological observations within sites but not independent replication of site-level microclimatic conditions.

Relative thallus area change between consecutive surveys was expressed as a log-response ratio, LRR = log((A_t_ + c)/(A_t−1_ + c)), where A_t_ and A_t−1_ are thallus areas at the current and previous survey, respectively, and c = 1 cm^2^ was added to accommodate zero values, corresponding approximately to the smallest thallus area that could be mapped consistently from the field photographs. Fourteen-day mean VPD was used as the primary hydroclimatic predictor, with 14-day mean air temperature included as a concurrent thermal covariate. Both predictors were standardised before analysis.

To match the scale at which microclimate was measured, the primary analysis was conducted at the site × survey interval level. For each interval, relative thallus area change was summarised as the median LRR across subplots within each site and modelled as a function of VPD and temperature.

We additionally examined the relationship at subplot level to assess whether the pattern was consistent with the primary analysis. A mixed-effects model with subplot identity as a random intercept yielded a singular fit with essentially zero random-intercept variance, and fixed-effect estimates were unchanged from the corresponding linear model. We therefore retained the linear model for this sensitivity analysis.

Additional sensitivity analyses assessed whether the VPD association depended on response aggregation, observations with very small previous thallus areas, response formulation, or site identity. The scale-matched analysis was repeated using mean rather than median LRR and with VPD as the sole predictor. The subplot-level LRR model was refitted after excluding observations below the 5th and 10th percentiles of previous thallus area. We also fitted an alternative area-state model in which log-transformed current thallus area was modelled as a function of log-transformed previous thallus area, VPD, and temperature. Finally, site identity was included as an additional fixed effect, and VPD–LRR relationships were visualised separately for each site.

Model assumptions were assessed using residual-versus-fitted plots, Q–Q plots, Cook’s distance and residual autocorrelation functions. Diagnostic plots shown in Figure S1 refer to the subplot-level LRR sensitivity model.

### 4.4. Cold-Season Thallus Dynamics and Frost Exposure

To assess whether cold-season thallus dynamics were consistent with direct frost damage, frost exposure was summarised for each site × survey interval from the 15 min temperature records and assigned to subplot-level observations from the corresponding site and interval. Cumulative duration below 0 °C within each complete 14-day interval was expressed as frost hours. Freeze–thaw frequency was calculated as the number of days per interval with minimum temperature below 0 °C and maximum temperature above 0 °C.

For frost-exposed intervals, relative thallus area change (LRR) was related to standardised cumulative frost duration using a linear model. As a complementary analysis, a binomial generalised linear model tested whether cumulative frost exposure was associated with the probability of negative thallus area change across all survey intervals with complete frost-exposure records, including intervals without frost.

Because frost exposure was measured at the site × survey interval level and shared among subplots within each site and interval, these analyses were treated as exploratory. They were used to assess whether thallus responses showed the direction expected under a simple frost damage hypothesis rather than as independently replicated tests of frost effects.

Sensitivity analyses considered alternative frost metrics, including the number of frost days and minimum temperature within the same 14-day interval. Freeze–thaw frequency was examined descriptively and in exploratory models because freeze–thaw days were few and unevenly distributed across intervals. Mixed-model comparisons, additional sensitivity analyses, influence checks and model diagnostics are reported in the Supplementary Materials (Figure S2).

### 4.5. Previous-Season Thallus State and Subsequent Reproduction

To examine whether subsequent reproduction was associated with previous-season thallus state, we derived a subplot-level index of thallus change during the 2023 growing season. Baseline thallus area was defined as the area recorded at the first survey in April 2023; where this measurement was unavailable, the first available measurement in 2023 was used. Previous-season thallus state was calculated as the log-ratio of maximum thallus area attained between April and October 2023 relative to baseline area, with an offset of 1 cm^2^ to accommodate zero values. This offset approximated the smallest thallus area that could be mapped consistently from the field photographs.

Reproduction in spring 2024 was quantified as both the presence and the cumulative number of newly recorded carpocephala per subplot. Because only eight subplots were available and reproductive outcomes were sparse, relationships with previous-season thallus state were treated as exploratory. We fitted a binomial model for reproductive presence/absence and a Poisson model for carpocephalum counts, using standardised previous-season thallus state and baseline thallus area as predictors. The fitted models showed numerical instability, and the count model showed overdispersion. We therefore did not interpret model coefficients inferentially; the models were used only to examine the direction of the relationships.

### 4.6. Reproductive Phenology and Carpocephalum Development

Reproductive phenology was assessed at each survey by recording reproductive structures and newly recorded carpocephala of the 2024 cohort. Spring reproductive expression was summarised as the occurrence of newly recorded carpocephala per survey interval from February to May 2024. Hydroclimatic context was described using mean VPD over the period preceding each survey. Fourteen-day means were used where complete records were available; 7-day means were used only for descriptive visualisation when complete 14-day windows were unavailable. VPD during intervals with and without newly recorded carpocephala was compared descriptively, and reproductive events were additionally summarised at the site × survey-date level.

Post-emergence development was assessed from repeated measurements of carpocephalum height. At each survey, up to ten carpocephala per site were measured where present. Height was summarised for each site and survey date using the 75th percentile of positive values as a descriptor of upper carpocephalum development. Seasonal height trajectories were interpreted descriptively in relation to hydroclimatic conditions.

Climate records were matched to reproductive survey dates using nearest-day matching within a ±1-day tolerance to account for minor timestamp offsets. No formal statistical inference was applied to reproductive phenology or carpocephalum development because observations were sparse, seasonally restricted and non-independent within sites. Carpocephala from the 2023 cohort were already present at the start of monitoring; emergence timing could therefore only be determined for newly recorded carpocephala of the 2024 cohort. Height measurements were retained across the full monitoring period and include the late development of the 2023 cohort as well as the 2024 cohort.

All analyses were conducted in R version 4.4.3. Climate data processing and date matching used dplyr, lubridate, readr, stringr, purrr and tibble; models and diagnostics used lme4, car, broom and broom.mixed; and figures were produced with ggplot2 and patchwork.

## Figures and Tables

**Table 1:** Linear model results for relative thallus area change, aggregated as the median log-response ratio at the site × survey interval scale, in relation to vapour pressure deficit and temperature. Predictors were standardised before analysis. *n* = 34 site × survey interval observations.

Predictor	Estimate β	SE	95% CI	*t*	*p*
VPD	−0.58	0.21	[−1.00, −0.16]	−2.81	0.008
Temperature	0.09	0.21	[−0.33, 0.51]	0.42	0.680

**Table 2:** Exploratory subplot-level linear model of relative thallus area change during frost-exposed cold-season intervals in relation to cumulative frost exposure. Frost exposure was measured at the site × survey interval scale and assigned to subplot-level observations. The predictor was standardised before analysis (mean = 0, SD = 1). Residual standard error = 0.66; *n* = 24 subplot-interval observations.

Predictor	Estimate β	SE	95% CI	*t*	*p*
Intercept	−0.12	0.13	[–0.40, 0.16]	−0.91	0.372
Frost hours	0.29	0.14	[0.01, 0.58]	2.11	0.046

## Data Availability

The raw data supporting the conclusions of this article will be made available by the authors on request.

## Supplementary Materials

The following supporting information can be downloaded at https://www.mdpi.com/article/10.3390/plants15182808/s1, Figure S1: Diagnostic plots for the linear model relating relative thallus area change to vapour pressure deficit and temperature; Figure S2: Diagnostic plots for the linear model relating relative thallus area change to cumulative frost exposure during cold-season intervals; Table S1: Comparison of linear and mixed-effects models for vegetative thallus area change; Table S2: Sensitivity analyses for the relationship between vapour pressure deficit and vegetative thallus area change; Table S3: Vegetative thallus area change model including site identity as a fixed effect; Table S4: Influence sensitivity for the exploratory cold-season frost model; Table S5: Sensitivity analysis of frost metrics for cold-season relative thallus area change; Table S6: Effects of cumulative frost exposure on the probability of negative relative thallus area change.

## References

[1] Tarascio M., Brancaleoni L., Cazzavillan A., Cutini M., Gerdol R., Abeli T. (2026). Photoperiod-Temperature Interactions in a Changing Climate: A Review of Plant Phenological Responses. J. Biogeogr..

[2] Turetsky M.R. (2003). The role of bryophytes in carbon and nitrogen cycling. Bryologist.

[3] Slate M.L., Antoninka A., Bailey L., Berdugo M.B., Callaghan D., Cárdenas M., Chmielewski M.W., Fenton N.J., Holland-Moritz H., Hopkins S. (2024). Impact of changing climate on bryophyte contributions to terrestrial water, carbon, and nitrogen cycles. New Phytol..

[4] Tuba Z., Proctor C., Csintalan Z. (1998). Ecophysiological responses of homoiochlorophyllous and poikilochlorophyllous desiccation tolerant plants: A comparison and an ecological perspective. Plant Growth Regul..

[5] Proctor M.C.F., Oliver M.J., Wood A.J., Alpert P., Stark L.R., Cleavitt N.L., Mishler B.D. (2007). Desiccation-tolerance in bryophytes: A review. Bryologist.

[6] Bisang I., Ehrlén J. (2002). Reproductive effort and cost of sexual reproduction in female *Dicranum polysetum*. Bryologist.

[7] Rydgren K., Okland R.H. (2003). Short-term costs of sexual reproduction in the clonal moss *Hylocomium splendens*. Bryologist.

[8] dos Santos W.L., Pôrto K.C., Pinheiro F. (2024). An Overview of Reproductive Allocation and Reproductive Costs in Bryophytes: Challenges and Prospects. Bot. Rev..

[9] Stark L.R. (2005). Phenology of patch hydration, patch temperature and sexual reproductive output over a four-year period in the desert moss *Crossidium crassinerve*. J. Bryol..

[10] Stark L.R., Oliver M.J., Mishler B.D., McLetchie D.N. (2007). Generational differences in response to desiccation stress in the desert moss *Tortula inermis*. Ann. Bot..

[11] McLetchie D.N., Stark L.R. (2006). Sporophyte and gametophyte generations differ in their thermotolerance response in the moss *Microbryum*. Ann. Bot..

[12] Milne J. (2001). Reproductive biology of three Australian species of *Dicranoloma* (Bryopsida, Dicranaceae): Sexual reproduction and phenology. Bryologist.

[13] De Oliveira S.M., Pôrto K.C. (2001). Reproductive phenology of the moss *Sematophyllum subpinnatum* in a tropical lowland forest of north-eastern Brazil. J. Bryol..

[14] Martins A., Fontinha S., Patiño J., Sim-Sim M. (2025). Seasonal effects on sex expression in dioecious bryophytes: Insights from an oceanic island region. Plant Biosyst..

[15] Schuster R.M., Longton R.E., Schuster R.M. (1983). Reproductive biology. New Manual of Bryology.

[16] Stark L.R. (2002). Phenology and its repercussions on the reproductive ecology of mosses. Bryologist.

[17] Zehr D.R. (1979). Phenology of selected bryophytes in southern Illinois. Bryologist.

[18] Longton R.E. (1979). Studies on growth, reproduction and population ecology in relation to microclimate in the bipolar moss *Polytrichum alpestre*. Bryologist.

[19] Longton R.E., Greene S.W. (1979). Experimental studies of growth and reproduction in the moss *Pleurozium schreberi* (Brid) Mitt. J. Bryol..

[20] Sundberg S. (2002). Sporophyte production and spore dispersal phenology in *Sphagnum*: The importance of summer moisture and patch characteristics. Can. J. Bot.-Rev. Can. Bot..

[21] Laaka-Lindberg S. (2005). Reproductive phenology in the leafy hepatic *Lophozia silvicola* Buch in southern Finland. J. Bryol..

[22] Barbé M., Fenton N.J., Caners R., Bergeron Y. (2017). Interannual variation in bryophyte dispersal: Linking bryophyte phenophases and weather conditions. Botany.

[23] Bengtsson F., Cronberg N., Villegas J.A.L., Siddique A., Stenberg P., Ekroos J. (2025). Rapid shifts in bryophyte phenology revealed by airborne eDNA. J. Ecol..

[24] Coe K., Carter B., Slate M., Stanton D. (2024). Moss functional trait ecology: Trends, gaps, and biases in the current literature. Am. J. Bot..

[25] Gollan J.R., Ashcroft M.B., Ramp D. (2013). Fine-grained climate data alters the interpretation of a trait-based cline. Ecosphere.

[26] Kemppinen J., Lembrechts J.J., Van Meerbeek K., Carnicer J., Chardon N.I., Kardol P., Lenoir J., Liu D.J., Maclean I., Pergl J. (2024). Microclimate, an important part of ecology and biogeography. Glob. Ecol. Biogeogr..

[27] During H.J. (1979). Life strategies of Bryophytes: A preliminary review. Lindbergia.

[28] Proctor M.C.F., Goffinet B., Shaw A.J. (2009). Physiological ecology. Bryophyte Biology.

[29] Zhang T.J. (2005). Influence of the seasonal snow cover on the ground thermal regime: An overview. Rev. Geophys..

[30] Wilson G., Green M., Brown J., Campbell J., Groffman P., Durán J., Morse J. (2020). Snowpack affects soil microclimate throughout the year. Clim. Change.

[31] Maruo F., Imura S. (2018). The effect of snow cover on the phenology of the moss *Racomitrium lanuginosum*. Bryologist.

[32] Maruo F., Imura S. (2020). Restriction of sexual reproduction in the moss *Racomitrium lanuginosum* along an elevational gradient. Ecol. Evol..

[33] Stark L.R., Brinda J.C., McLetchie D.N. (2009). An experimental demonstration of the cost of sex and potential resource limitation on reproduction in the moss *Pterygoneurum* (Pottiaceae). Am. J. Bot..

[34] Sagmo Solli I.M., Söderström L., Bakken S., Flatberg K.I., Pedersen B. (1998). Reproductive phenology of *Dicranum majus* in central Norway. J. Bryol..

[35] Maciel-Silva A.S., Válio I.F.M. (2011). Reproductive phenology of bryophytes in tropical rain forests: The sexes never sleep. Bryologist.

[36] Stark L.R. (2002). Skipped reproductive cycles and extensive sporophyte abortion in the desert moss *Tortula inermis* correspond to unusual rainfall patterns. Can. J. Bot.-Rev. Can. Bot..

[37] Duckett J.G., Pressel S., Kowal J. (2024). The biology of *Marchantia polymorpha* subsp. *ruderalis* Bischl. & Boissel. Dub in nature. Front. Plant Sci..

[38] Lockwood L.G. (1975). The Influence of Photoperiod and Exogenour Nitrogen-Containing Compounds on the Reproductive Cycles of the Liverwort *Cephalozia media*. Am. J. Bot..

[39] Lee L., Rosenstiel T.N., Eppley S.M.S. (2010). Variation of photoperiod response in moss gametangial formation. Bryologist.

[40] Longton R.E. (1988). Adaptations and strategies of polar bryophytes. Bot. J. Linn. Soc..

[41] Benassi M., Stark L.R., Brinda J.C., McLetchie D.N., Bonine M., Mishler B.D. (2011). Plant size, sex expression and sexual reproduction along an elevation gradient in a desert moss. Bryologist.

[42] Smith R.I.L., Convey P. (2002). Enhanced sexual reproduction in bryophytes at high latitudes in the maritime Antarctic. J. Bryol..

