# Supplementary material for: Hydroclimate, Thallus Dynamics and Multi-Seasonal Reproductive Phenology in Mannia triandra
Source: Plants (Basel). 2026 Sep 13;15(18):2808. doi: 10.3390/plants15182808 (PMC13610872; doi:10.3390/plants15182808)
Supplement: Supplementary file 1 [file plants-15-02808-s001.zip › plants-4533010-supplementary.pdf]

Supplement

Supplementary analysis S1. Mixed-model comparison.

To account for repeated measurements of the same subplots in the subplot-level sensitivity analysis, we fitted a linear mixed-effects model with subplot identity as a random intercept. The random-intercept standard deviation was estimated as zero, resulting in a singular fit. Fixed-effect estimates were unchanged relative to the corresponding linear model; therefore, the simpler linear model was retained for this sensitivity analysis.

Table S1. Comparison of linear and mixed-effects models for vegetative thallus area change. Fixed-effect estimates from the linear model (LM) and the corresponding linear mixed-effects model (LMM) with subplot identity as a random intercept. The random-intercept standard deviation in the LMM was estimated as zero, resulting in a singular fit. Predictors were standardised before analysis.

Model	Term	Estimate	SE	95% CI	*t*	Random-intercept SD	Residual SD	Singular fit
LM	Intercept	0.116	0.097	[−0.075, 0.307]	1.20	—	1.126	—
LM	VPD	−0.608	0.122	[−0.850, −0.366]	−4.97	—	1.126	—
LM	Temperature	0.139	0.122	[−0.102, 0.381]	1.14	—	1.126	—
LMM	Intercept	0.116	0.097	[−0.074, 0.305]	1.20	0.000	1.126	TRUE
LMM	VPD	−0.608	0.122	[−0.848, −0.368]	−4.97	0.000	1.126	TRUE
LMM	Temperature	0.139	0.122	[−0.100, 0.379]	1.14	0.000	1.126	TRUE

Supplementary analysis S2. Sensitivity to small previous thallus area and response formulation

Log-response ratios may become extreme when previous thallus area is very small. We therefore assessed whether the estimated VPD association was sensitive to such observations by refitting the subplot-level LRR model after excluding observations below the 5th and 10th percentiles of previous thallus area. To further test whether the result depended on the use of LRR as the response variable, we fitted an alternative area-state model in which log-transformed current thallus area was modelled as a function of log-transformed previous thallus area, VPD and temperature. Across all sensitivity analyses, VPD estimates remained negative and confidence intervals did not overlap zero (Table S2).

Table S2. Primary and sensitivity analyses for the relationship between vapour pressure deficit and vegetative thallus area change.

Models assessed whether the estimated association between vapour pressure deficit (VPD) and vegetative thallus area change was sensitive to the scale of analysis, extreme log-response ratios at small previous thallus areas, or the use of LRR as the response variable. The primary model aggregated relative thallus area change to the site × survey-interval scale, matching the scale at which microclimatic predictors were applied. Additional models were treated as sensitivity analyses. Predictors were standardised before analysis.

Model	Response	*n*	Term	Estimate	SE	95% CI	*t*	*p*
Subplot-level LRR sensitivity model	LRR	136	VPD	−0.608	0.122	[−0.850, −0.366]	−4.97	<0.001
Subplot-level LRR sensitivity model	LRR	136	Temperature	0.139	0.122	[−0.102, 0.381]	1.14	0.256
Primary site × interval median LRR model	Median LRR	34	VPD	−0.578	0.206	[−0.998, −0.159]	−2.81	0.008
Primary site × interval median LRR model	Median LRR	34	Temperature	0.086	0.206	[−0.334, 0.505]	0.42	0.680
Primaty site × interval median LRR model, VPD only	Median LRR	34	VPD	−0.526	0.161	[−0.854, −0.199]	−3.27	0.003
Site × interval mean LRR model	Mean LRR	34	VPD	−0.615	0.220	[−1.063, −0.167]	−2.80	0.009
Site × interval mean LRR model	Mean LRR	34	Temperature	0.141	0.220	[−0.307, 0.589]	0.64	0.526
LRR model excluding lowest 5% previous area	LRR	129	VPD	−0.588	0.102	[−0.789, −0.386]	−5.78	<0.001
LRR model excluding lowest 5% previous area	LRR	129	Temperature	0.206	0.103	[0.003, 0.410]	2.01	0.047
LRR model excluding lowest 10% previous area	LRR	122	VPD	−0.508	0.098	[−0.702, −0.314]	−5.19	<0.001
LRR model excluding lowest 10% previous area	LRR	122	Temperature	0.146	0.097	[−0.046, 0.338]	1.51	0.134
Alternative area-state model	log current area	136	log previous area	0.589	0.051	[0.489, 0.689]	11.62	<0.001
Alternative area-state model	log current area	136	VPD	−0.644	0.100	[−0.843, −0.446]	−6.42	<0.001
Alternative area-state model	log current area	136	Temperature	0.152	0.100	[−0.046, 0.351]	1.52	0.131

Supplementary analysis S3. Site-specific robustness

Table S3. Vegetative thallus area change model including site identity as a fixed effect. Predictors were standardised before analysis. Site S2 is shown relative to the reference site S1.

Predictor	Estimate	SE	95% CI	*t*	*p*
Intercept	0.299	0.142	[0.019, 0.579]	2.11	0.036
VPD	−0.699	0.132	[−0.960, −0.438]	−5.30	<0.001
Temperature	0.189	0.125	[−0.057, 0.436]	1.52	0.131
Site S2	−0.367	0.208	[−0.779, 0.045]	−1.76	0.080

Supplementary analysis S4. Model diagnostics.

Diagnostics for the subplot-level LRR sensitivity model did not indicate severe violations of model assumptions. Residuals showed moderate heteroscedasticity at higher fitted values, and Q–Q plots showed minor tail deviations. These deviations were consistent with extreme log-response ratios occurring when previous thallus area was small. Sensitivity analyses excluding observations with small previous thallus area and using an alternative area-state model showed that the negative VPD association was not driven solely by these observations or by the use of LRR as the response variable (Table S2). Influence diagnostics identified a small number of moderately influential observations, and residual autocorrelation was weak.

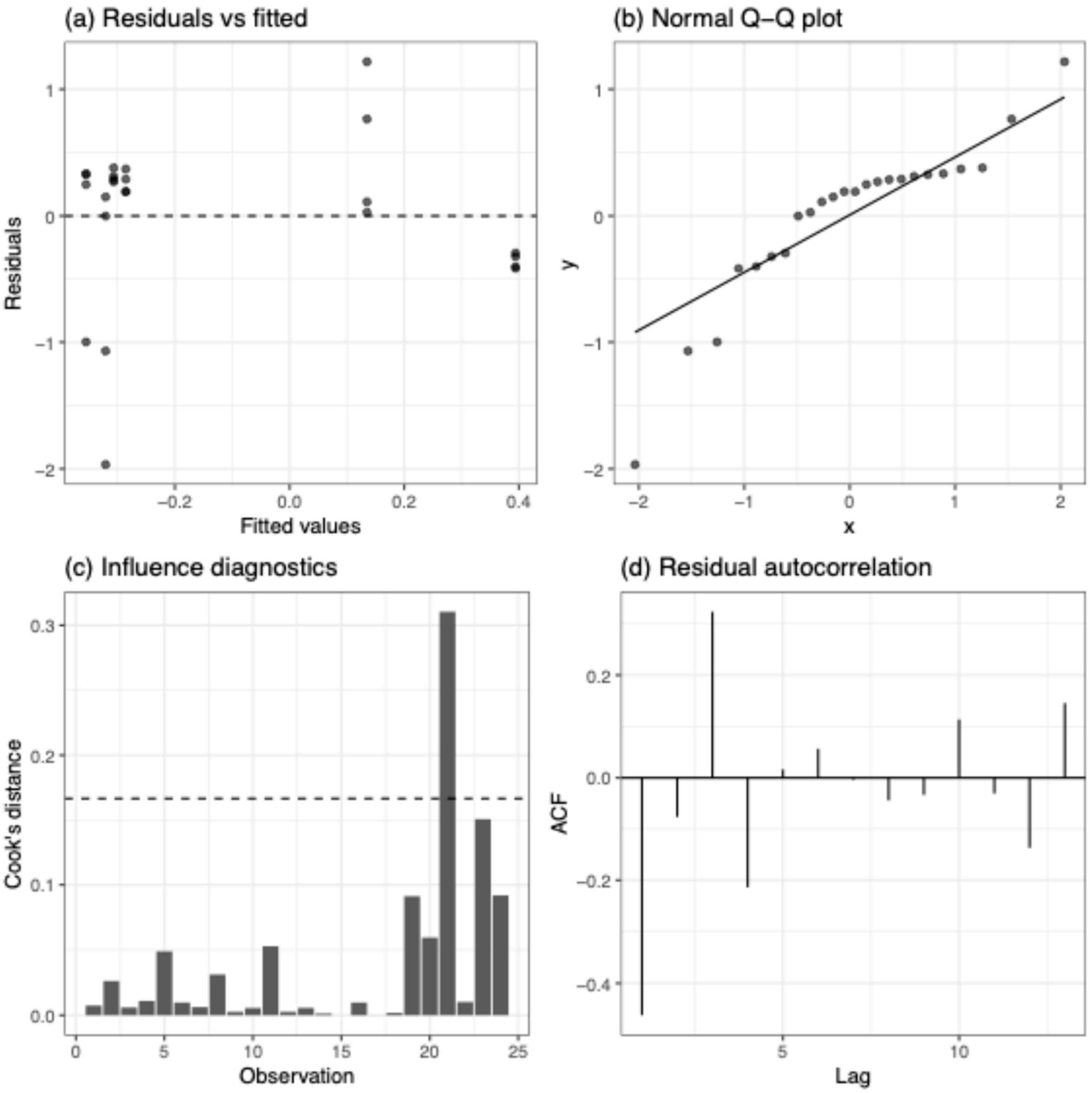

Figure S1. Diagnostic plots for the linear model relating relative thallus area change to vapour pressure deficit and temperature.
(a) Residuals versus fitted values,
(b) Q–Q plot of residuals,
(c) influence diagnostics (Cook's distance), and
(d) autocorrelation function (ACF) of residuals.

Supplementary analysis S5. Influence sensitivity for the exploratory cold-season frost model

Influence diagnostics identified a small number of observations with elevated Cook's distance. Refitting the exploratory cold-season frost model after excluding the most influential observation yielded a

similar positive frost estimate. Excluding the two most influential observations reduced the effect size and statistical support, but the direction of the estimate remained positive (Table S4). These sensitivity analyses supported the conservative interpretation used in the main text: the data did not support the predicted increase in thallus loss with longer frost exposure, while the positive estimate was influence-sensitive and was not interpreted as evidence for a beneficial effect of frost.
Because frost exposure was shared among subplots within each site × survey interval, these models were interpreted as exploratory despite the negligible subplot-level random-intercept variance.

Table S4. Influence sensitivity for the exploratory cold-season frost model.

Estimates are from linear models relating relative thallus area change to standardised cumulative frost exposure during frost-exposed cold-season intervals. Sensitivity models excluded the observations with the highest Cook's distance values.

Analysis	*n*	Estimate	SE	95% CI	*t*	*p*	Adjusted R^2
Exploratory frost model	24	0.291	0.138	[0.005, 0.577]	2.11	0.046	0.131
Excluding highest Cook's distance	23	0.229	0.108	[0.005, 0.453]	2.13	0.045	0.138
Excluding two highest Cook's distances	22	0.180	0.095	[−0.019, 0.380]	1.89	0.073	0.109

Alternative frost metrics

As a sensitivity analysis, we repeated the exploratory frost analysis using two alternative descriptors of cold exposure: the number of frost days within each 14-day interval and the minimum temperature recorded during the same interval. Frost days were defined as days with a minimum temperature below 0 °C. All continuous predictors were standardised before analysis.

Across these alternative metrics, model estimates did not support the expectation that colder or more prolonged frost exposure corresponded to greater thallus loss (Table S5). The results therefore led to the same qualitative interpretation as the cumulative frost-duration model: cold exposure metrics did not indicate greater thallus loss with increasing frost exposure.

Table S5. Sensitivity analysis of frost metrics for cold-season relative thallus area change.
Each predictor was tested in a separate linear model. Predictors were standardised (mean = 0, SD = 1).

Predictor	Estimate (β)	SE	95% CI	*t*	*p*
Frost hours	0.29	0.14	[0.01, 0.58]	2.11	0.046
Frost days	**0.35**	0.13	**[0.07, 0.62]**	**2.63**	0.015
Minimum temperature	−0.25	0.14	[−0.55, 0.04]	−1.81	0.084

Residual standard error = 0.63–0.68; *n* = 24 subplot-interval observations; microclimatic predictors were assigned at the site × survey-interval scale. Values reflect separate single-predictor models.

Model structure and random-effect sensitivity

Initial models included subplot identity as a random intercept to account for repeated observations within subplots. For the frost-duration analysis, the random-intercept variance was negligible and models showed singular fits. We therefore report the corresponding linear models in the main text, while using the random-effect models as a structural sensitivity check. The fixed-effect estimates and qualitative interpretation were unchanged by this simplification.

Collinearity among cold-season predictors

Cold-season temperature descriptors, including cumulative frost duration, frost days, minimum temperature and mean interval temperature, were strongly interrelated. We therefore did not interpret

models including multiple cold-season temperature descriptors as separating independent effects of frost duration and mean temperature. Instead, alternative frost metrics were used to assess whether the main interpretation was robust to different summaries of cold exposure.

Binary area-loss models

As a complementary analysis, we modelled whether cumulative frost exposure increased the probability of negative relative thallus area change. The response variable distinguished intervals with negative relative thallus area change from intervals with zero or positive change. This analysis included all survey intervals with complete 14-day frost-exposure summaries, including intervals without frost exposure. Binomial models were inspected for dispersion and influential observations. The results therefore led to the same qualitative interpretation as the cumulative frost-duration model: cold exposure metrics did not indicate greater thallus loss with increasing frost exposure.

Table S6. Effects of cumulative frost exposure on the probability of negative relative thallus area change.
Results are from a binomial generalised linear model. Frost exposure was standardised before analysis (mean = 0, SD = 1).

Predictor	Estimate (β)	SE	95% CI	*z*	*p*
Intercept	−0.25	0.19	[−0.63, 0.12]	−1.31	0.19
Frost hours	−0.12	0.19	[−0.55, 0.25]	−0.62	0.54

Residual deviance = 153.12; null deviance = 153.51; *n* = 112 subplot-interval observations; frost exposure was assigned at the site × survey-interval scale.

Freeze–thaw frequency

Freeze–thaw frequency was summarised as the number of days within a survey interval on which minimum temperature was below 0 °C and maximum temperature was above 0 °C. In the frost-exposed dataset, all retained intervals included at least one freeze–thaw day, and freeze–thaw frequency took only three values: 1, 3 and 8 days per 14-day interval. Because of this limited variation, freeze–thaw frequency was interpreted descriptively. Analyses including freeze–thaw frequency were therefore used only to describe whether the data suggested an additional frost-regime pattern beyond cumulative frost duration. No clear interpretable relationship between freeze–thaw frequency and relative thallus area change was detected.

Supplementary analysis S6. Model diagnostics for the exploratory cold-season frost model.

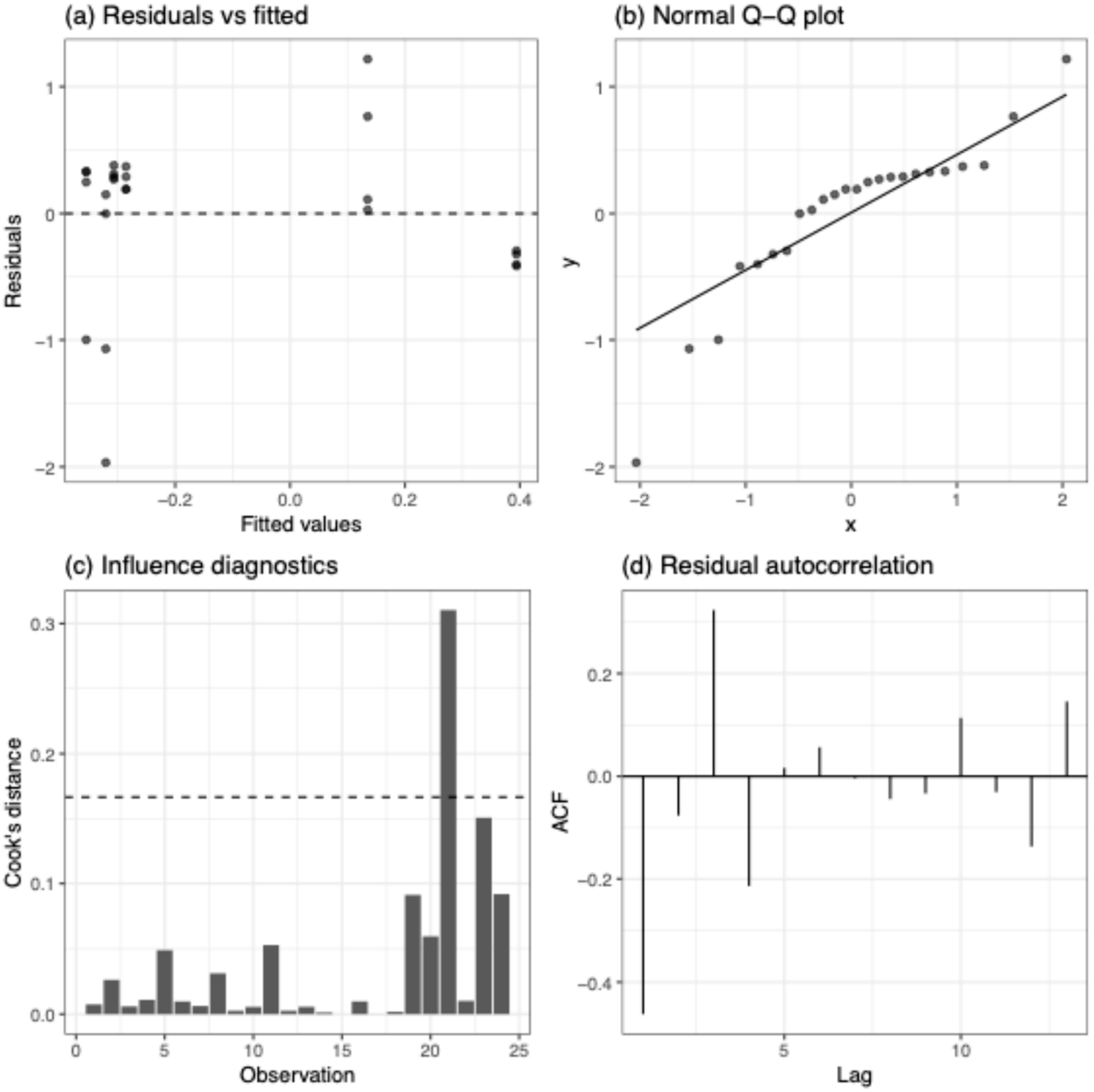

Figure S2. Diagnostic plots for the linear model relating relative thallus area change to cumulative frost exposure during cold-season intervals.
(a) Residuals versus fitted values, (b) Q–Q plot of residuals, (c) influence diagnostics using Cook's distance, and (d) autocorrelation function of residuals.
Overall, diagnostics indicated no strong violations of model assumptions. Influence diagnostics identified a small number of observations with elevated influence, which were evaluated in the influence-sensitivity analysis (Table S4).
